# Comprehensive Analysis of Allulose Production: A Review and Update

**DOI:** 10.3390/foods13162572

**Published:** 2024-08-17

**Authors:** Lei Wang, Yun Cui, Yujie Lu, Zongpei Zhao

**Affiliations:** 1 School of Grain Science and Technology, Jiangsu University of Science and Technology, Zhenjiang 212100, China; 2 Jiangsu Provincial Engineering Research Center of Grain Bioprocessing, Zhenjiang 212100, China; 3 School of Computer, Jiangsu University of Science and Technology, Zhenjiang 212100, China

**Keywords:** allulose, immobilization, directed evolution

## Abstract

Advancements in D-allulose production have seen significant strides in recent years, focusing on enzymatic conversion methods. Key developments include traditional immobilization techniques, the discovery of novel enzymes, directed evolution studies, and biosynthesis through metabolic pathway modification. Enzymatic conversion, particularly utilizing D-allulose 3-epimerase, remains fundamental for industrial-scale production. Innovative immobilization strategies, such as functionalized nano-beads and magnetic MOF nanoparticles, have significantly enhanced enzyme stability and reusability. Directed evolution has led to improved enzyme thermostability and catalytic efficiency, while synthetic biology methods, including phosphorylation-driven and thermodynamics-driven pathways, have optimized production processes. High-throughput screening methods have been crucial in identifying and refining enzyme variants for industrial applications. Collectively, these advancements not only enhance production efficiency and cost-effectiveness but also adhere to sustainable and economically viable manufacturing practices. The past five years have witnessed critical developments with significant potential impact on the commercial viability and global demand for allulose.

## 1. Introduction

Allulose, also known as psicose, is a rare sugar that has garnered substantial interest globally due to its low-calorie content and potential health benefits, particularly in managing diabetes and obesity. The rising consumer awareness of healthy eating habits has significantly increased the global demand for allulose [1,2,3,4]. North America, especially the United States, leads in production and technological advancements, with companies like Ingredion and Tate & Lyle playing pivotal roles. The adoption of allulose in various food and beverage products has been robust, further accelerating its demand [5]. In addition to the US, Japan and South Korea are also prominent markets, with companies like Matsutani Chemical and CJ CheilJedang pioneering the production and application of allulose [6].

**Figure 1:**
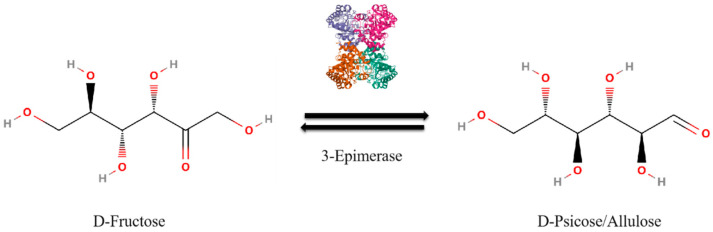
Schematic representation of reaction, where D-fructose is converted to D-allulose in the presence of 3-Epimerase.

The production of allulose involves sophisticated technologies, primarily enzymatic conversion and synthetic biology [7,8,9,10]. Enzymatic conversion, which utilizes specific enzymes to convert fructose into allulose, is widely preferred for its high yield and efficiency. The conversion of fructose to allulose involves the enzyme D-allulose 3-epimerase, which changes the configuration of the hydroxyl group at the third carbon atom of fructose, forming allulose (Figure 1) [11,12]. The conversion rate of fructose to allulose remains around 30% due to thermodynamic equilibrium. In the enzymatic reaction catalyzed by D-allulose 3-epimerase, the forward (fructose to allulose) and reverse (allulose to fructose) reactions reach a balance, stabilizing at this ratio [13,14,15,16,17,18]. This equilibrium reflects the free energy landscape of the reaction, where the energy difference between fructose and allulose is minimized. Thus, at equilibrium, the forward and reverse reaction rates are equal, limiting further conversion [14,19,20,21,22,23]. Overcoming this barrier requires innovative approaches to shift the equilibrium towards a higher allulose yield. Synthetic biology offers a promising solution to this equilibrium barrier. By genetically engineering microorganisms to optimize metabolic pathways, it is possible to enhance the efficiency of the conversion process. This approach can shift the equilibrium towards a higher yield of allulose, surpassing the natural thermodynamic limitations [8,24,25,26].

Significant progress has been made in the regulatory landscape for allulose. In 2019, the U.S. Food and Drug Administration (FDA) recognized allulose as Generally Recognized As Safe (GRAS), accelerating its adoption in the market [27]. Companies like Ingredion and Tate & Lyle have received GRAS status, allowing extensive marketing in the United States. The European Food Safety Authority (EFSA) is also evaluating allulose for use in the European Union, with recent indications suggesting a positive outcome [24].

China is emerging as a significant player in the allulose market, focusing on reducing sugar consumption and improving public health. Chinese companies are heavily investing in advanced production technologies and expanding capacities to meet both domestic and international demand. Baolingbao Biology Co., Ltd (Yucheng, China). has a production capacity of 5000 tons per year, while Shandong Sanyuan Biotechnology and CJ CheilJedang (Bingzhou, China) contribute significantly with capacities of 3500 and 4000 tons per year, respectively. These investments, amounting to millions of dollars, highlight their commitment to meeting the growing demand for allulose.

Regulatory support in China has been critical. The National Health Commission of China (NHC) recently approved allulose as a new food ingredient, facilitating its incorporation into a wider range of food products [28]. This regulatory support, combined with China’s increasing production capacity, is expected to significantly lower production costs and contribute more substantially to human health by providing a rare sugar alternative. Therefore, it is necessary to review the recent advancements in the production technology and research progress of allulose [6,17,29,30,31,32,33].

## 2. Recent Updates

### 2.1. Enzymatic Conversion

It is essential to discuss D-allulose 3-epimerase from four distinct perspectives: (1) Traditional methods involving immobilization and whole-cell fixed catalysis for large-scale production, (2) the discovery of novel enzymes, (3) directed evolution studies, and (4) biosynthesis through metabolic pathway modification. Enzymatic conversion remains a foundational technology for allulose production, particularly the isomerization of fructose to allulose utilizing specific enzymes like D-allulose 3-epimerase. Due to the progressive expansion of market demand, the identification of novel 3-epimerase enzymes and the application of immobilization and synthetic biology methods are critical strategies for advancing current industrial processes. Here, we have updated the research findings from the past five years, focusing on optimizing the activity and stability of these enzymes to enhance conversion efficiency (Table 1) [8,11,34,35].

#### 2.1.1. Immobilization

Sodium alginate as a food-grade immobilization method has been widely used in current allulose production, but new methods continue to emerge, offering significant advancements in enzyme stabilization and efficiency [36]. For instance, the study on D-allulose biotransformation from D-glucose, involving separation by Simulated Moving Bed Chromatography (SMBC) and purification by crystallization, demonstrated an impressive 98% purity of D-allulose with a 70% yield [12]. This integrated process leverages the high selectivity of SMBC to effectively separate D-allulose from the reaction mixture, followed by crystallization to achieve high purity. The ability to achieve such high purity and yield makes this process highly attractive for industrial-scale production, ensuring efficient conversion and separation.

Further innovation is seen in the use of functionalized polyhydroxyalkanoate (PHA) nano-beads as stable biocatalysts. These nano-beads were engineered to immobilize D-allulose 3-epimerase, resulting in enhanced enzyme stability and reusability. Specifically, the immobilized enzyme on PHA nano-beads retained 85% of its initial activity after 10 cycles of use, compared to the free enzyme, which lost 50% of its activity after just 5 cycles. This significant improvement in stability and reusability reduces production costs and makes the process more sustainable. The functionalized PHA nano-beads provide a durable platform that minimizes enzyme degradation, allowing for extended use in industrial processes.

Additionally, the two-step biosynthesis of D-allulose via a multienzyme cascade for the bioconversion of fruit juices demonstrated the potential of combining multiple enzymes in a single process [37]. This method involved the sequential use of fructose isomerase and D-allulose 3-epimerase, which improved the overall efficiency and yield of D-allulose production. The study reported a 75% conversion rate of D-fructose to D-allulose, with an overall yield of 60% from the initial fruit juice substrate. This efficient conversion process is particularly advantageous for utilizing complex substrates like fruit juices, optimizing each step of the enzymatic conversion. By employing a multienzyme cascade, this approach ensures a higher overall yield and reduces the need for additional processing steps, thereby streamlining the production process.

Lastly, the immobilization of D-allulose 3-epimerase into magnetic metal–organic framework (MOF) nanoparticles represents a cutting-edge approach to enzyme immobilization [38]. These magnetic MOF nanoparticles allowed for easy recovery and reuse of the enzyme, significantly enhancing the efficiency of the biocatalytic process. The study showed that the immobilized enzyme retained 90% of its activity after 15 cycles, compared to the free enzyme, which dropped to 40% activity after the same number of cycles. Additionally, the MOF nanoparticles provided a high surface area for enzyme immobilization, improving the enzyme’s stability and activity. The magnetic properties of the nanoparticles facilitated simple separation from the reaction mixture using a magnetic field, making the process highly practical for large-scale biocatalysis. This innovative method not only improves enzyme performance but also simplifies downstream processing, which is critical for industrial applications.

Collectively, these studies underscore significant advancements in immobilization techniques that enhance enzyme stability, reusability, and overall efficiency in D-allulose production. The use of SMBC and crystallization ensures high purity and yield, making it a robust method for industrial applications. Functionalized PHA nano-beads provide a sustainable and cost-effective platform for enzyme immobilization, extending enzyme lifespan and reducing costs. The multienzyme cascade approach optimizes the conversion process, particularly for complex substrates, enhancing overall production efficiency. Magnetic MOF nanoparticles offer a high-tech solution for enzyme recovery and reuse, improving both stability and activity. Together, these innovative methods pave the way for more sustainable and cost-effective industrial processes, ultimately contributing to the availability of healthier allulose alternatives and advancing the field of industrial biotechnology.

#### 2.1.2. Exploring Novel Industrial Enzymes

In order to explore novel industrial enzymes, numerous studies have focused on characterizing and enhancing D-allulose 3-epimerases, which are pivotal in producing rare sugars. The recombinant D-allulose 3-epimerase from *Agrobacterium* sp. ATCC 31749 revealed a critical interfacial residue, Lys-152, essential for catalytic activity, with mutations reducing activity by 70%, offering insights for enzyme engineering [39]. This finding is crucial as it provides a foundation for understanding how specific amino acid residues contribute to the enzyme’s function, thereby guiding future efforts to engineer more efficient and stable enzymes for industrial applications. A putative Dolichol Phosphate Mannose Synthase from *Bacillus* sp. exhibited a 35% conversion rate from D-fructose to D-allulose at 50 °C, maintaining 80% activity after 24 h [40]. This enzyme’s dual functionality, demonstrating both D-allulose 3-epimerase activity and the ability to convert D-fructose to D-allulose, could streamline industrial production processes by reducing the need for multiple enzymes. This stability and efficiency are crucial for industrial applications where continuous production processes are essential, offering potential cost savings and simplifying manufacturing workflows.

Another D-allulose 3-epimerase from *Bacillus* sp. demonstrated remarkable heat stability, with a half-life of 120 min at 70 °C, converting 30% of D-fructose to D-allulose at an optimal temperature of 65 °C [10]. The enzyme’s ability to function effectively at high temperatures makes it particularly suitable for industrial applications where heat stability is a critical factor. This property reduces the need for cooling systems, thereby lowering energy costs and enhancing the overall efficiency of the production process. The enzyme’s high temperature tolerance also opens up possibilities for its use in processes where maintaining lower temperatures would be impractical or cost-prohibitive.

A novel D-allulose 3-epimerase gene from the metagenome of a thermal aquatic habitat, expressed in *Bacillus subtilis*, achieved a 40% conversion rate from D-fructose to D-allulose at 60 °C. The enzyme retained 75% of its activity after 48 h of continuous operation, indicating its robustness and suitability for industrial use [13]. The utilization of whole-cell catalysis further demonstrates the enzyme’s potential for scalable D-allulose production, offering a cost-effective and efficient method for generating this rare sugar. This approach leverages the natural capabilities of whole cells to carry out complex biochemical transformations, simplifying the production process and potentially reducing costs associated with enzyme purification and stabilization.

The D-tagatose 3-epimerase from *Caballeronia fortuita* showed broad substrate specificity, converting 25% of D-fructose to D-allulose at 55 °C with an activity of 1.2 U/mg protein [41]. Its ability to produce various rare sugars from different substrates simplifies the production process and reduces costs. This versatility makes the enzyme from *Caballeronia fortuita* a valuable tool for industries looking to produce high-value sugar alternatives in a cost-effective manner. The enzyme’s broad substrate range and efficiency in converting multiple types of sugars highlight its potential for diverse industrial applications, including the production of rare sugars for food and pharmaceutical industries.

The enzyme from *Christensenella minuta* demonstrated a 32% conversion rate at 50 °C with a half-life of 150 min, highlighting its potential for sustainable sugar production [42]. The enzyme’s efficiency and stability at moderate temperatures make it suitable for various industrial applications, including food and beverage production, where mild processing conditions are preferred to maintain product quality. The robustness of this enzyme under practical industrial conditions suggests it could be integrated into existing production systems with minimal modification, enhancing the sustainability and efficiency of sugar production processes.

Directed evolution techniques were employed to enhance the thermostability of the D-allulose 3-epimerase from *Clostridium cellulolyticum* H10 [43]. The improved enzyme exhibited a half-life increase from 30 min to 180 min at 65 °C and achieved a 45% conversion rate of D-fructose to D-allulose. These enhancements make the enzyme more viable for industrial applications, where prolonged stability at elevated temperatures is often required. The use of directed evolution to improve enzyme properties highlights the potential for tailoring enzymes to meet specific industrial needs, thereby optimizing production processes. This approach underscores the power of modern biotechnological methods to create enzymes with enhanced properties tailored for specific industrial applications.

A hyperthermostable l-ribulose 3-epimerase from *Labedella endophytica* retained 90% of its activity after 24 h at 80 °C, with an optimal temperature of 75 °C [21]. The enzyme’s ability to convert 28% of D-fructose to D-allulose under these conditions underscores its potential for high-temperature industrial processes. Its robustness makes it a promising candidate for applications where extreme conditions are encountered, such as in the production of certain chemicals and pharmaceuticals. The enzyme’s high thermostability means it can be used in processes that require sustained high temperatures, potentially reducing the need for cooling and stabilization steps and thereby lowering overall production costs.

The enzyme from *Novibacillus thermophilus* showed optimal activity at 70 °C, retaining 85% of its activity after 12 h at this temperature [44]. It achieved a 38% conversion of D-fructose to D-allulose, demonstrating its effectiveness for industrial-scale sugar synthesis. The enzyme’s efficiency and thermal stability make it particularly advantageous for D-allulose production in processes that demand prolonged enzyme activity.

A food-grade expression system in *Corynebacterium glutamicum* facilitated the conversion of cane molasses to D-allulose with a conversion efficiency of 42%, retaining 80% of its activity after 24 h [36]. This system enhances the enzyme’s production and application in food industries, providing a cost-effective and scalable method for producing D-allulose from readily available raw materials like cane molasses. This innovation has significant implications for the food industry, where there is a growing demand for healthier allulose alternatives. By using a food-grade expression system, this approach ensures that the produced D-allulose meets safety and regulatory standards for use in food products.

Similarly, the D-tagatose 3-epimerase from *Sinorhizobium* sp. achieved a 35% conversion rate of D-fructose to D-allulose at 55 °C, with 85% of its activity retained after 24 h [45]. The enzyme’s robust performance under these conditions suggests its applicability in industrial settings, particularly where continuous operation is necessary.

Finally, the recombinant D-allulose 3-epimerase from *Thermoclostridium caenicola* showed a conversion rate of 38% from D-fructose to D-allulose at 60 °C, retaining 80% of its activity after 24 h [22]. These enzymes collectively demonstrate significant potential for integration into large-scale production systems, where their consistent performance over extended periods is essential for maintaining process efficiency and reducing costs, paving the way for more widespread use of D-allulose in various products. These studies collectively highlight significant advancements in enzyme characterization, thermostability improvements, and efficient biocatalytic applications, driving the industrial production of D-allulose and other rare sugars forward. By focusing on enzymes with high stability and activity under industrial conditions, these studies pave the way for more efficient and cost-effective production processes, ultimately contributing to the availability of healthier allulose alternatives.

**Table 1:** Recent typical research on 3-Epimerase properties.

No	Source	Temperature (°C)	pH	Metal Ions	Half-Life (min)	Equilibrium Ratio (D-Allulose to D-Fructose)	Specific Activity * (U/mg)	Kcat/Km (mM^−1^min^−1^)	References
1	*Agrobacterium* sp.	55–60	7.5–8.0	Co^2+^	267(55 °C)	30.0%	253.0	19.5	[23]
2	*Bacillus* sp.	40	7.5	Co^2+^	25 (50 °C)	30.7%	185.7	-	[40]
3	*Bacillus* sp. KCTC 13219	55	8.0	Mn^2+^	36,000 (50 °C), 1320 (55 °C)	28.5%	127.2	-	[10]
4	*Bacillus subtilis*	80	7.0	Co^2+^	9900 (60 °C), 3240 (70 °C), 49 (80 °C)	31.0%	1.1	-	[13]
5	*Caballeronia fortuita*	65	7.5	Co^2+^	63 (60 °C)	29.5% (65 °C)	270.0	432.6	[41]
6	*Christensenella minuta* DSM 22607	50	6.0	Ni^2+^	40 (50 °C)	30% (50 °C)		124.0	[42]
7	*Clostridium cellulolyticum* H10	70	8.0	Co^2+^	537 (65 °C), 1573 (60 °C) A107P/D281G/C289R	27.5%	295.5	-	[43]
8	*Labedella endophytica*	80	6.0	Ni^2+^	2262 (60 °C), 540 (65 °C), 276 (70 °C)	-	110.7	-	[21]
9	*Novibacillus thermophilus*	70	7.0	Co^2+^	846.3 (40 °C) 539.2 (50 °C), 47.8 (60 °C)	28.3%	146.0	1354.9	[44]
10	*Paenibacillus senegalensis*	55	8.0	Mn^2+^	140 (60 °C)	30% (55 °C) 30% (60 °C)	25.2	39.0	[36]
11	*Pirellula* sp. SH-Sr6A	60	7.5	Co^2+^	360 (60 °C)	31.4%	-	-	[37]
12	*Rhodopirellula baltica*	60	8.0	Mn^2+^	52 (60 °C)	-	37.6	43.3	[46]
13	*Sinorhizobium* sp.	50	8.0	Mn^2+^	-	25.3%	-	118.2	[45]
14	*Staphylococcus aureus*	70	8.0	Mg^2+^	122.4 (70 °C)	28.3%	38.4	-	[47]
15	*Thermoclostridium caenicola*	65	7.5	Co^2+^	120 (55 °C)	28.0%	-	4.9	[22]

* Substrate: D-fructose.

#### 2.1.3. Direct Evolution Study

Recent advancements in engineering D-allulose 3-epimerase (DAEase) have enhanced its thermostability, catalytic efficiency, and acid resistance, which is crucial for industrial D-allulose production [22]. Utilizing directed evolution—a method of iterative mutation and selection—has proven that single amino acid mutations can significantly improve enzyme properties. When single mutations fail, neutral mutations often stabilize the protein, enabling subsequent beneficial changes or altering “promiscuous” functions to evolve new capabilities. These insights from directed evolution have been applied to DAEase, leading to improved enzyme performance and more efficient, sustainable industrial production of low-calorie D-allulose [48].

Through directed evolution, mutants of DAEase from *Clostridium cellulolyticum* H10, such as D281G and C289R, demonstrated significantly increased half-lives at 65 °C, with the triple mutant A107P/D281G/C289R showing a 58.85-fold increase in half-life and improved thermostability by 14.39 °C [43]. Similarly, computational tools identified beneficial mutations in *Thermoclostridium caenicola* DAEase, resulting in the four-point mutant Var3 with enhanced rigidity and stability due to new hydrogen bonds and optimized electrostatic charge distribution [49]. A sequence- and structure-based approach identified the highly thermostable TtDAE from *Thermogutta terrifontis*, with the M4 variant exhibiting a 5.12-fold increase in catalytic efficiency and a significantly higher melting temperature [48]. Proline residue substitutions in *Clostridium bolteae* DAEase, particularly the double mutant Cb-51P/89P, led to a 2.32-fold increase in half-life at 55 °C [50]. Furthermore, engineering efforts for acid resistance produced the triple mutant M3 (I114R/K123E/H209R) with a 3.36-fold increase in activity and a 10.6-fold increase in acid resistance at pH 4.5, making it suitable for functional juice production [51]. Rational design of DAEase from *Halanaerobium congolense* resulted in the Y7H/C66L/I108A and Y7H/C66L/I108A/R156C/K260C mutants, with the latter showing a half-life of 5.2 h at 70 °C and increased melting temperature by 6.5 °C [52]. These studies highlight the synergy between computational predictions and experimental validation, revealing that enhancing enzyme stability and activity through targeted genetic modifications is crucial for industrial applications. The use of proline substitutions and disulfide bridges, combined with B-factor analysis, underscores the importance of understanding protein dynamics for effective enzyme engineering [53]. The development of acid-resistant variants further expands the application of DAEase to acidic environments, showcasing the enzyme’s versatility and potential. Collectively, these research efforts address the current limitations of DAEase and set a precedent for future studies aimed at further optimizing these biocatalysts, paving the way for broader applications in biotechnology and beyond.

#### 2.1.4. Synthetic Biology Method

Traditional whole-cell catalysis typically employs engineered *Corynebacterium glutamicum* or *Bacillus subtilis*, and new metabolic pathways are continually being explored [8]. Recent research on ATP involvement in metabolic catalysis is noteworthy and may bring significant changes to synthetic biology methods. Synthetic biology methods can enhance the allulose conversion rate and break the equilibrium between fructose and allulose. The production of D-allulose through synthetic biology has garnered significant interest due to its health benefits and economic value. This analysis compares three prominent methods: phosphorylation-driven production, thermodynamics-driven production, and the use of metabolically engineered *Escherichia coli*, highlighting their integrated pathways and efficiencies [34].

Phosphorylation-driven production stands out for its coupling with an ATP regeneration system. In this method, D-glucose is converted to D-allulose through a series of enzymatic steps. Key enzymes include hexokinase, which phosphorylates D-glucose to glucose-6-phosphate, and glucose-6-phosphate isomerase, which converts it to fructose-6-phosphate. Fructose-6-phosphate is further converted to allulose-6-phosphate by D-tagatose 3-epimerase and finally dephosphorylated to yield D-allulose. The ATP regeneration system involves creatine kinase, which helps maintain high ATP levels, enhancing the overall efficiency and yield of the production process [54].

**Figure 2:**
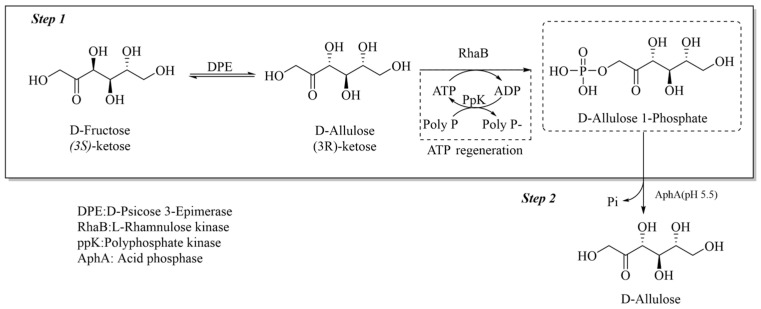
The conversion of D-fructose into D-allulose coupling with phosphorylation method.

**Figure 3:**
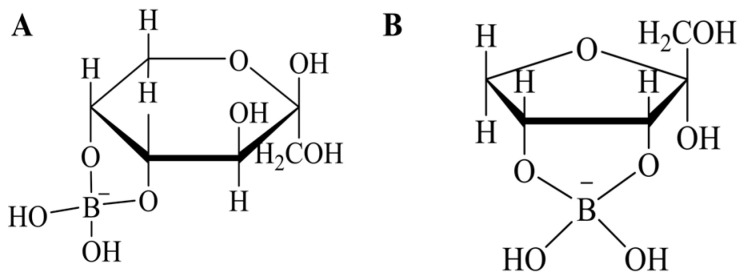
Chemical structures of the sugar–borate complexes. (**A**) Fructose as β-d-fructopyranose cis-C-4,5 diol borate. (**B**) Psicose as α-d-furanopsicose cis-C-3,4 diol borate.

A highly efficient pathway for synthesizing D-allulose from D-fructose uses D-psicose epimerase and L-rhamnulose kinase integrated with an ATP regeneration system via polyphosphate kinase (Figure 2) [55]. This method achieves an impressive 99% conversion rate by optimizing key reaction conditions, significantly reducing ATP consumption to 10% of the theoretical amount and employing a fed-batch mode to mitigate polyphosphate inhibition. Traditionally, boric acid has been used to drive the D-allulose synthesis reaction forward by forming complexes with fructose, thereby shifting the equilibrium towards allulose production (Figure 3). However, while boric acid significantly increases the conversion yield by leveraging its high binding affinity to D-allulose, it poses environmental and health risks due to its toxicity and difficulty in its removal from the final product. In contrast, the enzymatic approach leverages the natural catalytic efficiency of enzymes and an ATP regeneration cycle to sustainably propel the reaction without harmful additives. This advancement underscores the potential of metabolic engineering to enhance enzymatic efficiency and economic feasibility in biochemical production. The successful integration of ATP regeneration not only reduces costs but also eliminates the need for boric acid, making the process more environmentally friendly [56].

Conversely, the thermodynamics-driven method optimizes the natural energy dynamics of enzymatic reactions, converting inexpensive starch into D-allulose. This method involves the breakdown of starch into glucose units using α-amylase and glucoamylase [20]. The glucose is then isomerized to fructose by glucose isomerase. Finally, D-fructose is converted to D-allulose by D-allulose 3-epimerase. The process is conducted in vitro, allowing for precise control over reaction conditions, and leverages the thermodynamic properties of the enzymes to drive the reactions, minimizing the need for external energy inputs.

Metabolically engineered *Escherichia coli* presents a different strategy. Here, microbial cells are genetically modified to facilitate the conversion of D-fructose to D-allulose through a phosphorylation-dephosphorylation cycle [54]. The engineered *E. coli* strain harbors genes encoding fructokinase, which phosphorylates D-fructose to fructose-6-phosphate, and a D-tagatose 3-epimerase that converts fructose-6-phosphate to allulose-6-phosphate. The allulose-6-phosphate is then dephosphorylated by a specific phosphatase to produce D-allulose. This method harnesses the biological machinery of *E. coli*, allowing for a sustainable and potentially scalable production process.

Evaluating cost-effectiveness, the thermodynamics-driven approach excels due to its reliance on inexpensive starch and minimal external energy requirements. The phosphorylation-driven method may incur higher costs due to the need for ATP and the sophisticated regeneration system required to maintain it. The *E. coli*-based method’s cost-effectiveness is influenced by the expenses associated with genetic engineering and microbial cultivation, although advancements in microbial engineering could mitigate these costs over time [57].

Scalability is another critical factor. The microbial method using *E. coli* offers significant advantages due to the ease of microbial culture and the potential for further genetic modifications. This approach can be scaled up relatively easily, provided the metabolic pathways are optimized for high yield and low by-product production. Both the phosphorylation-driven and thermodynamics-driven methods are also scalable but require more complex bioreactors and precise control systems to maintain optimal reaction conditions.

Yield and purity are crucial determinants of the viability of these production methods. The phosphorylation-driven method, with its efficient use of ATP, can achieve high yields of D-allulose, reported up to 90% conversion efficiency. The thermodynamics-driven method provides good yields by optimizing reaction conditions to favor the conversion of starch to D-allulose, achieving up to 85% yield in laboratory conditions. The *E. coli* method’s yield depends heavily on the efficiency of the engineered pathways, with current studies reporting yields around 70–80%, but ongoing advancements in synthetic biology are expected to enhance these yields significantly.

Each method for D-allulose production presents unique advantages and challenges. The choice of method depends on specific production requirements, such as cost, scalability, energy efficiency, and yield. Continuous research and innovation in synthetic biology and metabolic engineering will be key to realizing the full potential of these methods, making D-allulose production more sustainable and economically viable on an industrial scale. By moving away from boric acid and similar methods, this strategy presents a greener, safer alternative for large-scale production of D-allulose, aligning with the growing demand for environmentally responsible manufacturing practices.

### 2.2. High-Throughput Screening Methods

High-throughput screening methods are critical in the identification and optimization of novel D-allulose 3-epimerase (DAEase) variants, which are essential for the efficient industrial production of D-allulose. Recent advancements in screening methodologies have significantly contributed to enhancing the thermostability and catalytic efficiency of these enzymes. Two prominent high-throughput screening approaches, as documented in recent reports, exemplify the progress in this field.

One approach, recently reported, utilizes a Continuous Spectrophotometric Assay (CSA) for the efficient determination of D-allulose concentrations in reaction mixtures [58]. This assay employs the nicotinamide adenine dinucleotide (NADH)-dependent ribitol dehydrogenase (KpRD), which specifically reduces D-allulose while oxidizing an equimolar amount of NADH to NAD⁺ simultaneously. This dual-enzyme system enables rapid high-throughput screening of DAEase variants with improved thermostability and catalytic activity. For instance, the DAEase variant MT exhibited a 2.2-fold increase in half-life at 60 °C, underscoring its potential for industrial application. Moreover, the activity of SfDAE was notably enhanced in the presence of Co^2+^, Mg^2+^, and Mn^2+^, with respective increases of 1.24-, 1.43-, and 1.35-fold. This CSA approach offers a robust tool for the rapid evaluation and screening of desired DAEase variants, facilitating their adaptation for industrial processes. Another method, also recently reported, involves a dual-enzyme system combined with the Seliwanoff reagent, which differentiates between D-allulose and D-fructose. This method comprises three key steps. Initially, D-fructose is converted to D-allulose by DAE until equilibrium is reached, with the remaining fructose content indicating enzyme activity. Subsequently, xylose isomerase (XI) is introduced to convert the residual fructose to glucose [43]. The presence of active DAE results in lower fructose and, consequently, less glucose production compared to control reactions with inactive DAE. Finally, the Seliwanoff test quantifies the concentration of ketoses, including D-allulose and D-fructose, through absorbance measurement at 480 nm. This method was employed to screen for mutants with enhanced thermostability, yielding variants such as H56R, Q277R, H56R/Q277R, and H56R/Q277R/S293R. These variants demonstrated significant improvements in thermostability and catalytic efficiency, with the triple mutant H56R/Q277R/S293R showing a 7.1 °C increase in T50 and a 9.47-fold improvement in half-life at 60 °C compared to the wild-type enzyme. These two high-throughput screening methods—recently reported Continuous Spectrophotometric Assay and the dual-enzyme system with the Seliwanoff test—provide powerful and efficient tools for the discovery and optimization of DAEase variants. These advancements are pivotal for enhancing the industrial applicability of D-allulose production processes, ensuring higher efficiency and stability of the enzymes involved.

## 3. Summary

Technological advancements in allulose production are not only focused on improving efficiency but also on addressing environmental and economic concerns. Sustainable production methods, such as the use of renewable feedstocks and green chemistry principles, are being incorporated into the production processes. For instance, using agricultural by-products as substrates for microbial fermentation reduces waste and adds value to otherwise low-value materials.

Economic analyses of these advanced production methods have shown that optimizing enzyme performance and utilizing integrated production systems can significantly reduce the cost of allulose production. These cost reductions are critical for making allulose a competitive alternative to traditional sweeteners in the market.

The rapid advancements in enzymatic conversion, synthetic biology, and integrated production systems are revolutionizing allulose production. These technologies not only enhance the efficiency and scalability of allulose production but also align with global trends toward sustainable and economically viable manufacturing processes.

Future research should continue to focus on improving enzyme stability and activity through advanced protein engineering techniques. Additionally, exploring new microbial hosts and optimizing their metabolic pathways can further enhance microbial production systems. The integration of continuous production systems and the development of more efficient downstream processing techniques will be crucial for the commercial viability of allulose.

Collaboration between industry and academia is essential for translating these technological advancements into practical applications. By leveraging the expertise and resources of both sectors, it will be possible to overcome the remaining challenges in allulose production and fully realize its potential as a healthy and sustainable sugar alternative.

## 4. Conclusions

In conclusion, the global trend in allulose production is marked by a shift towards sustainable and efficient bio-based methods. Key players are investing in advanced technologies to optimize production processes, with China making notable contributions through significant investments in R&D and technological advancements. The collaboration between industry and academia is essential for overcoming production challenges and meeting the increasing global demand for allulose. Recent regulatory approvals, particularly by the FDA and anticipated approvals by EFSA, further enhance the market potential of allulose, signaling a bright future for this innovative sugar substitute. Additionally, the recent approval by China’s National Health Commission significantly boosts the domestic market potential for allulose, supporting its integration into a wide range of food products and aligning with global health trends.

## Data Availability

No new data were created or analyzed in this study. Data sharing is not applicable to this article.
